# Age and Racial Inequities in Telemedicine Internet Support Among Nephrology Outpatients During the COVID-19 Pandemic

**DOI:** 10.1016/j.xkme.2021.05.001

**Published:** 2021-07-05

**Authors:** Nwamaka D. Eneanya, Taylor L. Stallings, Jordan Shaffer, Michael E. Konu, Jordana B. Cohen, Sarah J. Schrauben, Jonathan J. Hogan, Deirdre L. Sawinski

**Affiliations:** 1 Renal-Electrolyte Division, Perelman School of Medicine, University of Pennsylvania, Philadelphia, Pennsylvania; 2 Palliative and Advanced Illness Research Center, Perelman School of Medicine, University of Pennsylvania, Philadelphia, Pennsylvania; 3 Center for Clinical Epidemiology and Biostatistics, Perelman School of Medicine, University of Pennsylvania, Philadelphia, Pennsylvania; 4 Leonard Davis Institute of Health Economics, University of Pennsylvania, Philadelphia, Pennsylvania

To the Editor:

In response to the need for social distancing and safety concerns during the COVID-19 pandemic, many outpatient clinics across the country shifted from primarily conducting in-person clinic assessments to telemedicine visits. However, a recent study demonstrated racial and age inequities in use of telemedicine during the pandemic.^1^ Specifically, older and racial minority patients seen in primary and specialty medicine clinics were less likely to complete video visits. The reasons for these inequities remain unclear and may be owing to difficulties with using telemedicine video technologies. In this cross-sectional analysis of adult patients scheduled for nephrology telemedicine visits, we assessed overall telemedicine video access and internet support needs. This quality improvement project was conducted in accordance with the University of Pennsylvania Institutional Review Board Quality/Performance Improvement Project Policy and did not require formal research ethics committee review or informed consent.

We contacted all patients scheduled for visits at general and transplant nephrology clinics associated with the University of Pennsylvania between May and August 2020 (after local shutdown mandates had been implemented). We assessed access to video telemedicine using modified questions from Pew Research Center’s “Mobile Technology and Home Broadband 2019” report.^2^ Additionally, we ascertained internet support needs with the following item: “If you use the internet regularly, do you do it yourself or with help?” We obtained demographics from the electronic medical chart.

A total of 298 patients completed the survey. Baseline characteristics of patients who we reached were similar to those who we could not reach (Table 1). Additionally, compared to patients who completed general nephrology visits, transplant patients were younger (mean age, 52 years [SD ± 12] vs 57 years [SD ± 16], *P* < 0.01, Table S1) and less often spoke English (90 % vs 97 %, *P* < 0.01, Table S1).

**Table 1:** Baseline Characteristics for Responders and Nonresponders

Variable	Responders (N = 298)	Nonresponders (N = 376)	*P*
Mean age, years (± SD)	55 (± 15)	55 (±15)	0.75
Sex, n (%)			0.98
Male	167 (56)	211 (56)	
Female	131 (44)	165 (44)	
Race, n (%)			<0.01
White	138 (46)	160 (43)	
Black	138 (46)	160 (43)	
Asian	10 (3)	19 (5)	
Other	8 (3)	34 (9)	
Unknown	4 (1)	3 (1)	
Ethnicity, n (%)			0.14
Hispanic	13 (4)	15 (4)	
Non-Hispanic	282 (95)	361 (96)	
Unknown	3 (1)	0	
Marital status, n (%)			0.34
Married	145 (49)	197 (52)	
Not married	153 (51)	179 (48)	
Primary language, n (%)			0.77
English	283 (95)	360 (96)	
Non-English	11 (4)	13 (3)	
Unknown	4 (1)	3 (1)	

Percentages may not add up to 100 owing to rounding.

The majority of patients reported accessing video telemedicine with either a smartphone, iPad/tablet, or computer (98 %, Table S2). Those with access were younger than those without access (mean age, 55 years [SD ± 15] vs 68 years [SD ± 15], *P* = 0.04). Additionally, patients with access more often spoke English compared to those without access (95 % vs 83 %, *P* < .01). In analyses adjusted for sex, race, marital status, language, and visit type, older age was significantly associated with having less access to telemedicine (adjusted odds ratio [aOR] 0.91 [95 % CI, 0.83-0.99], Table S2).

Among 285 patients who answered the internet support question (Table 2), most reported using the internet by themselves (79 %) as opposed to needing help (21 %). Patients who needed help were older than those who did not (mean age, 60 years [SD ± 13] vs 53 years [SD ± 15], *P* < 0.01). In analyses adjusted for age, sex, race, marital status, language, and visit type, age ≥65 years (aOR 2.24 [95 % CI, 1.15-4.35]), and non-White race (aOR, 2.33 [95 % CI, 1.24-4.38], Table 2) were significantly associated with needing help with accessing the internet.

**Table 2:** Telemedicine Internet Support Needs

Variable	Use Alone (N = 226)	Needs Help (N = 59)	*P*	Unadjusted Odds Ratio (95 % CI)	Adjusted Odds Ratio^a^ (95 % CI)
Mean age, years (± SD)	53 (± 15)	60 (± 13)	<0.01	-	-
Age, n (%)					
Age <65 years (ref)	166 (73)	36 (61)	0.06	-	-
Age ≥65 years	60 (27)	23 (39)		1.77 (0.97-3.22)	2.24 (1.15-4.35)
Sex, n (%)			0.25		
Male (ref)	130 (58)	29 (49)		-	-
Female	96 (42)	30 (51)		1.40 (0.79-2.49)	1.50 (0.82-2.74)
Race, n (%)			0.02		
White (ref)	114 (50)	20 (34)		-	-
Non-White	112 (50)	39 (66)		1.99 (1.09-3.61)	2.33 (1.24-4.38)
Marital status, n (%)			0.68		
Not married (ref)	118 (52)	29 (49)		-	-
Married	108 (48)	30 (51)		1.13 (0.64-2.01)	1.20 (0.65-2.22)
Primary language, n (%)			0.11		
English (ref)	218 (96)	54 (92)		-	-
Non-English	8 (4)	5 (8)		2.52 (0.80-8.02)	2.41 (0.71-8.16)
Visit type, n (%)			0.15		
Transplant (ref)	73 (32)	25 (42)		-	-
Nephrology	153 (68)	34 (58)		0.65 (0.36-1.17)	0.55 (0.29-1.06)

Missing data for 13 individuals.

a Multivariable analyses adjusted for age, sex, race, marital status, language, and visit type.

In this survey of patients scheduled for nephrology telemedicine visits, we confirmed that older patients had less access to video telemedicine compared to younger patients.^1^ We also demonstrated that older and non-White patients were more likely to need help with accessing the internet. These findings are especially important given the higher prevalence of kidney disease among racial minorities and older populations,^3^ and also underscore the importance of explicitly assessing patients’ telemedicine capabilities in order to deliver effective and quality care.

Incorporating video telemedicine into clinical practice is an attractive and convenient option for many patients.^4^^,^^5^ However, older patients with kidney disease have lower eHealth literacy, which results in lower proficiency in effectively finding, evaluating, and using information via health technologies.^6^ This could be owing to having less experience with various telemedicine modalities as well as having a higher prevalence of disabilities.^7^ Additionally, racial minority individuals may find navigating video telemedicine challenging given that they more often access the internet exclusively through smartphones (where telemedicine platform interfaces may differ from a computer or tablet) compared to White individuals.^2^ For some older and racial minority patients, structural barriers such as financial restraints or lack of social support at home may greatly influence internet access and ultimately determine whether care occurs virtually versus in the office or emergency room.^8^^,^^9^

Given these data, ambulatory nephrology practices utilizing telemedicine video visits during the COVID-19 pandemic and beyond should employ strategies to assess patient readiness at the time a visit is scheduled. These include asking screening questions about patients’ social support and their preferred device for internet access. Targeted training to patients who require more support may also help streamline virtual video visits. Alternatively, clinicians may weigh the pros and cons of seeing certain patients in person or using the phone if barriers to video visits cannot be circumvented. In this time of crisis, we are obliged to improve the use of video telemedicine for our most at-risk patient populations by identifying key modifiers of health inequities and implementing feasible solutions.
