# Supplementary material for: Age and Racial Inequities in Telemedicine Internet Support Among Nephrology Outpatients During the COVID-19 Pandemic
Source: Kidney Med. 2021 Jul 5;3(5):868–70. doi: 10.1016/j.xkme.2021.05.001 (PMC8497035; doi:10.1016/j.xkme.2021.05.001)
Supplement: Supplementary File (PDF) — Tables S1-S2. [file mmc1.pdf]

Table S1. Baseline characteristics for general nephrology and transplant visits[#]

**Variable**	**General nephrology (N=197)**	**Transplant (N=101)**	**P value**
Mean age, years (± SD)[*]	57 (± 16)	52 (±12)	< 0.01
Sex, n (%)			0.18
Male	105 (53)	62 (61)	
Female	92 (47)	39 (39)	
Race, n (%)			0.33
White<br>Black<br>Asian<br>Other<br>Unknown	91 (46)<br>95 (48)<br>4 (2)<br>4 (2)<br>3 (2)	47 (47)<br>43 (43)<br>6 (6)<br>4 (4)<br>1 (1)	
Ethnicity, n (%)			0.93
Hispanic<br>Non-Hispanic<br>Unknown	8 (4)<br>187 (95)<br>2 (1)	5 (5)<br>95 (94)<br>1 (1)	
Marital Status, n (%)			0.83
Married<br>Not married	95 (48)<br>102 (52)	50 (50)<br>51 (50)	
Primary language, n (%)			< 0.01
English<br>Non-English<br>Unknown	192 (97)<br>5 (3)<br>0	91 (90)<br>6 (6)<br>4 (4)	

[#]Percentages may not add up to 100 due to rounding

Table S2. Telemedicine Access# (N = 298)

Variable	No Telemedicine (N=6)	Telemedicine (N=292)	P value
Mean age, years (± SD)*	68 (± 15)	55 (±15)	0.04
Sex, n (%)			0.76
Male	3 (50)	164 (56)	
Female	3 (50)	128 (44)	
Race, n (%)			0.88
White<br>Black<br>Asian<br>Other<br>Unknown	2 (33)<br>4 (67)<br>0<br>0<br>0	136 (47)<br>134 (46)<br>10 (3)<br>8 (3)<br>4 (1)	
Ethnicity, n (%)			0.83
Hispanic<br>Non-Hispanic<br>Unknown	0<br>6 (100)<br>0	13 (4)<br>276 (95)<br>3 (1)	
Marital Status, n (%)			0.45
Married<br>Not married	2 (33)<br>4 (67)	143 (49)<br>149 (51)	
Primary language, (%)			< 0.01
English<br>Non-English<br>Unknown	5 (83)<br>0<br>1 (17)	278 (95)<br>11 (4)<br>3 (1)	
Visit type, n (%)			0.98
General nephrology<br>Transplant	4 (67)<br>2 (33)	193 (66)<br>99 (34)	

#Telemedicine access defined as having access to smartphone or Ipad or computer

*Age significantly associated with having any type of telemedicine (aOR = 0.91, 95% CI: 0.83-0.99) in analyses adjusted for race, marital status, language, and visit type.
